# Effect of acupuncture of sphenopalatine ganglion for the treatment of allergic rhinitis: A protocol for a systematic review and meta-analysis

**DOI:** 10.1097/MD.0000000000023286

**Published:** 2020-11-20

**Authors:** PeiYu Xiong, Tao Yuan, Lu Xu, Bo Jia

**Affiliations:** College of Basic medicine, Chengdu University of Traditional Chinese Medicine, Chengdu, PR China.

**Keywords:** acupuncture, allergic rhinitis, protocol, sphenoid ganglion, systematic review

## Abstract

**Background:**

Allergic rhinitis is an allergic disease of the nasal mucosa mediated by IgE after the body is exposed to allergens. Acupuncture of sphenoid ganglion is a new technique developed by Professor Li Xinwu in the 1860 s to treat allergic rhinitis the efficacy of acupuncture on the sphenopalatine ganglion in the treatment of AR has been clinically verified, but a systematic review and meta-analysis of them is lacking. Our purpose is to evaluate the efficacy and safety of acupuncture on the sphenopalatine ganglion in the treatment of AR.

**Methods:**

We will search 8 electronic databases, including: Web of Science, PubMed, Cochrane Library, Embase, CNKI, CBM, Wanfang, VIP, WHO ICTRP, ChiCTR, Clinical Trials, Grey Literature Database. The literature search, screening and extraction will be carried out independently by 2 researchers. When the opinions are not uniform, it depends on the opinion of the third researcher. We will use RevmanV.5.3 to perform a fixed-effect meta-analysis on the date of clinical homogeneity studies, and the level of evidence will pass GRADE method.

**Results:**

This systematic review and meta-analysis will put a high-quality synthesis of the efficacy and safety of acupuncture of sphenoid ganglion treatment in AR.

**Conclusion:**

The review will provide a comprehensive basis for the treatment of AR patients with acupuncture on the sphenopalatine ganglion.

**Ethics and dissemination:**

Since this article does not involve patient privacy, ethical approval is not required.

**Trial registration number:**

INPLASY2020100067.

## 1 Introduction

Allergic rhinitis (AR) is a non-infectious disease of the nasal mucosa mainly mediated by serum immunoglobulin E (IgE) after the body is exposed to allergens, Its main clinical manifestations are paroxysmal sneezing, nasal itching, nasal congestion, runny nose, etc., and can be accompanied by symptoms such as eyes.^[1]^ According to its statistics, AR global incidence rate of about 25% to 35%, and increased year by year, even in Japan can be as high as 44.2%.^[2]^ AR not only seriously affects the daily life and work of patients, but also causes a certain economic burden, and becomes one of the potential risk factors of asthma attack.^[3]^ Therefore, effective treatment of AR is significant. At present, clinical treatment is mainly based on glucocorticoids, antihistamines, anti-leukotrienes and other drugs, but long-term use has certain side effects.

Acupuncture of sphenopalatine ganglion is a new technique developed by Professor Li Xinwu in the 1860 s for the treatment of allergic rhinitis. According to clinical reports, acupuncture of sphenopalatine ganglion is effective in treating various types of rhinitis up to 94%, and the cure rate is 70.6%.^[4]^ Since then, some doctors have used this method to treat allergic rhinitis, and the curative effect has been confirmed. The needle entry point of the sphenopalatine ganglion is located between the zygomatic arch and the coronoid of the mandible,^[5]^ close to the Xiaguan point.^[6]^ Acupuncture usually uses 0.35x60 mm filigree needles to pierce directly from here to the pterygopalatine fossa that is where the sphenopalatine ganglia is located to improve nasal ventilation and reduce intranasal gland secretion.^[7]^ As a result, the purpose of this study is to systematically review the existing literature to evaluate the effectiveness and safety of acupuncture of the sphenopalatine ganglion in the treatment of allergic rhinitis.

## 2 Methods

### 2.1 Study registration

The protocol was registered on INPLASY (registration number. INPLASY2020100067). The protocol will be reported in the systematic review and meta-analysis of the preferred project report (PRISMA-P^[5]^) statement guidelines.

### 2.2 Eligibility and exclusion criteria

#### 2.2.1 Types of study

All relevant RCTs published in English and Chinese on acupuncture sphenopalatine ganglion for AR can be included. Non-randomized controlled trials, reviews, case reports, experimental studies, expert experience and duplicate publications will be beexcluded.

#### 2.2.2 Types of participants

Participants in different age ranges with AR can be included in the study without restricting nationality, sex, race, occupation or education. Patients with vasomotor rhinitis, non-allergic rhinitis with eosinophilia syndrome, infectious rhinitis, hormonal rhinitis, drug-induced rhinitis, aspirin intolerance triad, cerebrospinal rhinorrhea were excluded.

#### 2.2.3 Types of interventions

The Study focus on clinical trials of acupuncture sphenopalatine ganglion in the treatment of AR, and the results will provide clinicians with consultation and advice. Therefore, the experimental group treated only with acupuncture and without any combination of other drugs and treatment will be included, regardless of the method of needle insertion, duration and frequency.

#### 2.2.4 Type of comparators

Studies of control groups will be treated with treatment and other interventions (e.g., drugs, conventional acupuncture).

#### 2.2.5 Types of outcome measurements

Main outcomes: The nasal symptom score will be evaluated as the primary outcome. The nasal symptom score (TNSS): sneezing, runny nose, itchy nose, nasal congestion and ocular symptom.

Additional outcomes: Quality of life questionnaire for nasal conjunctivitis (RQLQ); Visual analogue scale (VAS).

### 2.3 Search strategy

The details were adjusted according to the specific databases including Chinese Biomedical Literature (CBM), the China National Knowledge Infrastructure Database (CNKI), Wangfang Database (WF), Chinese Scientific Journal Database (VIP), Web of Science, Embase, PubMed, Cochrane Library, the World Health Organzation International Trials Registry Platform (WHO ICTRP), Chinese Clinical Trial Register (ChiCTR), Clinical Trials, Grey Literature Database. No limitation on language or publication type's restriction will be applied. Table 1 Search strategy for the PubMed database.

**Table 1:** Search strategy for the PubMed database.

#1	allergic rhinitis [Title/Abstract]
#2	anaphylactic rhinitis [Title/Abstract]
#3	#1 OR #2
#4	Sphenopalatine Ganglion [Title/Abstract]
#5	Xinwu Point [Title/Abstract]
#6	;Die’e Point [Title/Abstract]
#7	#4 OR #5 OR #6
#8	acupuncture [Title/Abstract]
#9	#3AND #7 AND #8
#10	Allergic Rhinitis [MeSH Terms]
#11	Acupuncture therapy [MeSH Terms]
#12	(#3 OR #10) AND #11
#13	#12 OR #9
#14	Clinical [Title/Abstract]
#15	Trial [Title/Abstract]
#16	#14 AND #15
#17	clinical trials as topic [MeSH Terms]
#18	clinical trial [Publication Type]
#19	random^∗^[Title/Abstract]
#20	random allocation [MeSH Terms]
#21	therapeutic use [MeSH Subheading]
#22	#16 OR #17 OR #18 OR #19 OR #20 OR #21
#23	#22 AND #13

### 2.4 Date collection and analysis

#### 2.4.1 Studies selection

**Figure 1:**
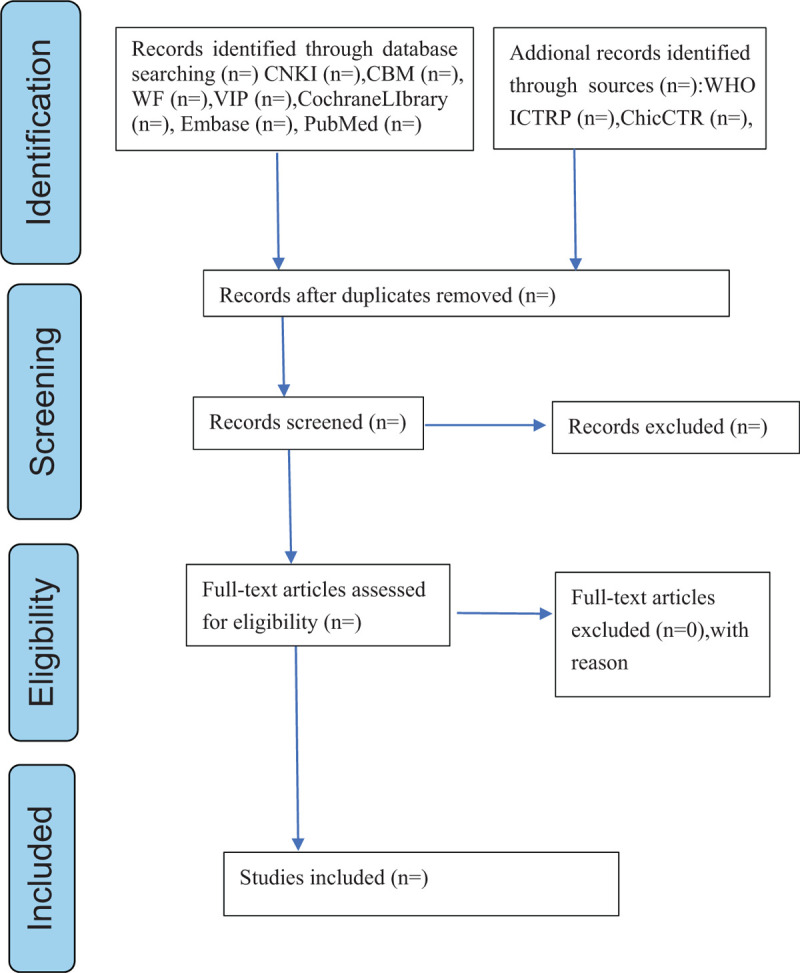
Flow diagram of the study selection process.

Literature retrieval, screening and data extraction were conducted by 2 researchers (YT, XL) independently through a standardized eligibility form. In case of disagreement, a third-party assessor (XPY) shall be consulted to assist judgment, and the missing information shall be supplemented by contacting the author. The general information of the selected articles will be extracted, including first author, country, year of publication, study design, duration of follow-up, duration of disease, sample size, detailed intervention, control treatment and the like. When the data of articles are sufficient or ambiguous, one of the authors will contact the original author to request detailed and additional information by e-mail or telephone. All selection processes will be represented by the PRISMA flow chart (Fig. 1).

#### 2.4.2 Data extraction and management

According to the indicators required for screening, we have produced an EXCEL form that meets the relevant requirements for data input and analysis before data extraction. Two independent reviewers (YT, XL) will extract data from eligible studies and enter the following information in the data extraction sheet. When the data can not be extracted through discussion to reach a consensus, the decision will be made by the third-party reviewers (XPY). The table will extract the main information of the selected article, including title, first author, country/region, publication year, study design, follow-up time, disease duration, sample size, detailed interventions, control treatment, primary/secondary outcome indicators, adverse reactions and so on. The above information will be cross-checked by 2 reviewers. If there is a dispute, they will directly contact the corresponding author of the article to check the information.

#### 2.4.3 Assessment of risk of bias in included studies

In the literature evaluation, we will use the Risk of Bias in Systematic reviews (ROBIS)^[1]^ tool provided by the Cochrane Handbook to conduct a rigorous quality assessment of the literature. Evaluation content includes: random allocation method, concealment of distribution, blindness of method, completeness of result data, selective reporting of research results, other sources of bias, etc. Through the evaluation of the above content quality, it is divided into 3 levels: “low risk of bias”, “uncertain risk of bias”, and “high risk of bias”. When the 2 reviewers cannot reach a consensus, it can be handed over to the third-party reviewer for decision.

#### 2.4.4 Measure of treatment effect

Using the relative risk ratio (RR) of 95% confidence interval dichotomous outcomes assessment, and continuous analysis of data mean difference (MD) with 95% CI or standard CD (SCD).

#### 2.4.5 Dating with missing date

When possible, we will choose to analyze data based on intentions. If data is missing or incomplete presence, we will contact the original investigator to obtain missing data results. If this data is not available in the present analysis will depend on the existing data.

#### 2.4.6 Assessment of heterogeneity

According to the Cochrane Handbook, we will choose the Chi-Squared test and the *I*^2^ test to analyze whether there is heterogeneity in the research. When *P* < 1, *I*^2^ > 50%, it is considered that there is heterogeneity between experiments, and random effects model can be used for analysis. On the contrary, there is no heterogeneity between the data, and the fixed effects model (FEM) comprehensive data is selected 2.4.4. Strategy for date synthesis

#### 2.4.7 Assessment of reporting biases

When the included study sample size is greater than 10, we will use a funnel chart to assess publication bias. If the funnel chart is unevenly distributed, it indicates that there is publication bias, and vice versa.

#### 2.4.8 Date synthesis

RevMan5.3.5 will be used for all statistical analyses. Based on the heterogeneity levels of the included studies, the fixed-effects model (*I*^2^ < 50%) or random-effects model (*I*^2^ ≥ 50%) will be selected. The dichotomous data will be analyzed by RR with 95% CIs, while the continuous data will be analyzed by MD/SMD with 95% CIs. The meaningful heterogeneity will be explained by any additional assessment included sensitivity analysis or subgroup analysis depended on the data.

#### 2.4.9 Subgroup analysis

If necessary, subgroup analysis will be performed based on different types of acupuncture therapy, participant characteristics, and outcome measures.

#### 2.4.10 Sensitivity analysis

When the subgroup analysis is unsatisfactory, we will use sensitivity analysis to evaluate the robustness of the main results. Then meta-analysis reorganizes and merges the data, and finally compares with the previous results.

#### 2.4.11 Grading the quality of evidence

The quality of the evidence will be graded and assessed according to the “Grades of Recommended Assessment, Designation and Evaluation (GRADE)” standard.^[8]^ All the evidence of the quality of the results is divided into 4 levels (high, medium, low, and very low) based on the results of 5 indicators (study limitations, inconsistent, indirect, uncertainty, publication bias).

## 3 Discussion

Allergic rhinitis is a non-infectious chronic inflammatory disease of the nasal mucosa mainly mediated by IgE after the body is exposed to allergens. It is one of the most common respiratory allergic diseases in clinical practice. Although AR is not life-threatening, it causes long-term distress to patients lives and work due to its repeated or persistent disease. The incidence of AR in China is increasing year by year, especially in children and adolescents. In 2011, the average prevalence of AR in children in 8 central cities in China was 9.8%.^[9]^ In the United States, the incidence of AR is 14% for adults and 13% for children.^[10]^ According to the survey, the number of AR patients worldwide currently exceeds 500 million.^[11]^ The incidence of AR is closely related to factors such as environment, genetics, and nutrition. The incidence of AR is closely related to factors such as environment, genetics, and nutrition. Therefore, the prevention and treatment principles of AR include environmental control, drug therapy, and specific immunotherapy and so on. Clinical treatment of AR includes intranasal and oral antihistamines, intranasal and oral decongestants, intranasal and oral corticosteroids, intranasal anticholinergics, and oral leukotriene receptor antagonists.^[12]^

In China, acupuncture is one of the common treatments for allergic diseases. The efficacy of acupuncture in the treatment of allergic rhinitis has been clinically affirmed.^[13,14]^ Compared with traditional acupoints, acupuncture on the sphenopalatine ganglion for allergic rhinitis has the characteristics of simple point selection, no dialectical matching of acupoints, and fewer acupuncture parts. It has obvious effects in both long-term and short-term treatment. At present, a large number of clinical studies have also proved that acupuncture at the sphenopalatine ganglion can effectively reduce the clinical symptoms of AR.^[15,16]^ However, there is still a lack of evidence-based medicine to prove whether acupuncture on the sphenopalatine ganglion is effective in improving the clinical symptoms of AR patients. Therefore, this program will analyze and summarize the effectiveness and safety of acupuncture on the sphenopalatine ganglion in the treatment of AR, and provide medical staff with more effective information. Therefore, this program will analyze and summarize the effectiveness and safety of acupuncture on the sphenopalatine ganglion in the treatment of AR, and provide medical staff with more effective information and better guide clinical treatment.

## Author contributions

**Conceptualization:** PeiYu Xiong, Bo Jia.

**Data curation:** Tao Yuan, Lu Xu.

**Formal analysis:** PeiYu Xiong, Lu Xu.

**Funding acquisition:** Bo Jia.

**Methodology:** PeiYu Xiong.

**Project administration:** Bo Jia.

**Supervision:** Tao Yuan.

**Writing – original draft:** PeiYu Xiong, Bo Jia.

**Writing – review & editing:** PeiYu Xiong, Bo Jia.
